# The now and future of ChatGPT and GPT in psychiatry

**DOI:** 10.1111/pcn.13588

**Published:** 2023-09-11

**Authors:** Szu‐Wei Cheng, Chung‐Wen Chang, Wan‐Jung Chang, Hao‐Wei Wang, Chih‐Sung Liang, Taishiro Kishimoto, Jane Pei‐Chen Chang, John S. Kuo, Kuan‐Pin Su

**Affiliations:** ^1^ College of Medicine China Medical University Taichung Taiwan; ^2^ Mind‐Body Interface Laboratory (MBI‐Lab) and Department of Psychiatry China Medical University Hospital Taichung Taiwan; ^3^ Department of Information Management Chia‐Nan University of Pharmacy & Science Tainan Taiwan; ^4^ Department of Electronic Engineering Southern Taiwan University of Science and Technology Tainan Taiwan; ^5^ Internet of Things Laboratory (IOT Lab) Medical and Intelligent Technology Research Center (MIT Center), Southern Taiwan University of Science and Technology Tainan Taiwan; ^6^ Department of Family Studies and Child Development Shih Chien University Taipei Taiwan; ^7^ Department of Psychiatry Beitou Branch, Tri‐Service General Hospital, National Defense Medical Center Taipei Taiwan; ^8^ Hills Joint Research Laboratory for Future Preventive Medicine and Wellness Keio University School of Medicine Tokyo Japan; ^9^ Neuroscience and Brain Disease Center China Medical University Taichung Taiwan; ^10^ Graduate Institute of Biomedical Sciences China Medical University Taichung Taiwan; ^11^ An‐Nan Hospital, China Medical University Tainan Taiwan

**Keywords:** artificial intelligence, ChatGPT, deep learning, GPT, informatics and telecommunications in psychiatry

## Abstract

ChatGPT has sparked extensive discussions within the healthcare community since its November 2022 release. However, potential applications in the field of psychiatry have received limited attention. Deep learning has proven beneficial to psychiatry, and GPT is a powerful deep learning‐based language model with immense potential for this field. Despite the convenience of ChatGPT, this advanced chatbot currently has limited practical applications in psychiatry. It may be used to support psychiatrists in routine tasks such as completing medical records, facilitating communications between clinicians and with patients, polishing academic writings and presentations, and programming and performing analyses for research. The current training and application of ChatGPT require using appropriate prompts to maximize appropriate outputs and minimize deleterious inaccuracies and phantom errors. Moreover, future GPT advances that incorporate empathy, emotion recognition, personality assessment, and detection of mental health warning signs are essential for its effective integration into psychiatric care. In the near future, developing a fully‐automated psychotherapy system trained for expert communication (such as psychotherapy verbatim) is conceivable by building on foundational GPT technology. This dream system should integrate practical ‘real world’ inputs and friendly AI user and patient interfaces *via* clinically validated algorithms, voice comprehension/generation modules, and emotion discrimination algorithms based on facial expressions and physiological inputs from wearable devices. In addition to the technology challenges, we believe it is critical to establish generally accepted ethical standards for applying ChatGPT‐related tools in all mental healthcare environments, including telemedicine and academic/training settings.

The recent emergence of the artificial intelligence (AI) chatbot, ChatGPT, has attracted enormous attention and sparked discussion about its applications in medicine and healthcare.^1^, ^2^, ^3^, ^4^ However, discussion about ChatGPT's potential uses in psychiatry is quite limited. We aim to provide insights into the current state of ChatGPT applications in the field of psychiatry and envision a potential future of digital mental health care *via* integration and advances in GPT technology.

## Understanding GPT and ChatGPT

The Generative Pre‐Trained Transformer (GPT) is a language model developed by OpenAI, Inc. ChatGPT is a GPT‐based chatbot that is trained to generate human‐understanding texts from inputs (prompts).

As its name suggests, GPT is a Transformer‐based AI that can create new contents (generative) based on its training data (pre‐training). GPT was designed for natural language processing (NLP) tasks that involve two important aspects: (1) analyzing and determining the meaning of a sentence (natural language understanding), and (2) generating new sentences based on inputs (natural language generation).

NLP classical models are rule‐based and results in outputs of limited answers based on a set of encoded rules. Thus, they are inflexible and struggle to adapt to the dynamic and diverse nature of language. In contrast, the ChatGPT generative models employing machine learning approaches to auto‐learn language patterns results in outputs of more‐contextual answers that fit forward‐and‐backward sequential meanings in sentences. They are thus more flexible and outperform classical rule‐based NLP models.

**Fig. 1:**
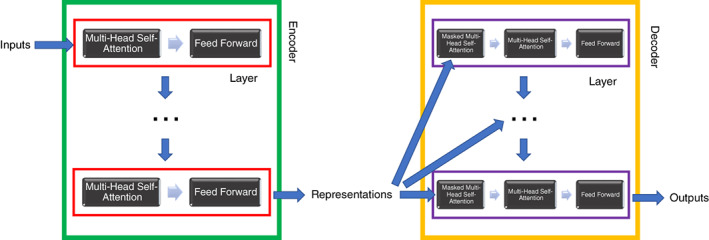
Simplified structure of the transformer. The encoder (green box) receives inputs for processing through 6 identical layers (red box) into a sequence of continuous representations. Each layer has a sublayer of a multi‐head self‐attention and a sublayer of a feed forward network. The decoder (orange box) receives and processes these representations through another 6 identical layers (purple box) into outputs. The layers in the decoder are similar to the ones in the encoder but have an additional masked multi‐head self‐attention sublayer to receive encoder‐generated representations. This sublayer grants the model auto‐regressive property: the model only depends on prior words in a sentence to predict words at a specific position. Positional information of words are encoded and passed separately in the model (not shown in the figure). The GPT model only utilized the decoder structure of the Transformer (Transformer‐decoder‐only structure).

The origin of NLP models can be traced back to 1949, when Weaver's memorandum^5^ first introduced the idea of machine translation (MT). Early NLP programs focused heavily on MT, but models with more diverse functions were also developed. ELIZA^6^ and PARRY^7^ were examples of psychiatry‐related systems. Systems developed before 1990 were rule‐based and heavily influenced by language theories. The revolutionary advance of introducing statistical models occurred in the early 1990s, followed by a paradigm shift to machine learning. Starting in the early 2000s, the prevalent use of deep learning laid the foundation for modern NLP models. An initial breakthrough was the 2003 introduction of the pioneer neural language model by Bengio *et al*.^8^ This model was a ‘one‐hidden layer’ feed‐forward neural network and probably the earliest to utilize the ‘word embedding’ method. Another technology jump occurred with the exponential increase in computational power and collection of large‐scale datasets in the 2010s, leading to effective implementation of recurrent neural networks (RNN) and long short‐term memory (LSTM). These advanced network structures provide impactful advantages in prediction or classification with sequential datasets. Since then, two breakthroughs form the foundation for GPT. First, novel algorithms like sequence‐to‐sequence learning (2014),^9^ attention (2015),^10^ and self‐attention (2017)^11^ were proposed and greatly enhanced the performance of generative NLP models (the “G” in GPT). The second breakthrough was the emergence of innovative ‘word embedding’ techniques: a type of word representation that records the importance, usage rates, and user‐specified meanings of words as numeric data representing the similar values of words with similar meanings. The first of such techniques was Word2Vec^12^ in 2013. Compared with previous methods, these techniques were more efficient and enabled large‐scale training on exponentially huge data sets. The concept of large pre‐trained language models was then introduced in 2016.^13^ (the “P” in GPT) Along with NLP technical and conceptual advancements, the Transformer, GPT's core architect, was published in 2017.^11^ This innovative architect enabled NLP models to record and process relevance between words in sentences. The original Transformer architect consists of an encoder and a decoder (Fig. 1). The encoder receives inputs for processing and transformation through six identical layers into a sequence of continuous representations. Then the decoder processes these representations through another six identical layers to generate outputs. Both the layers in the encoder and the decoder have a sublayer of a ‘multi‐head self‐attention’ and a sublayer of a fully connected ‘feed‐forward’ network. Moreover, each layer in the decoder has an additional sublayer of ‘masked self‐attention’ that only depends on prior words in a sentence to predict words at a specific position (auto‐regression).

OpenAI first published GPT in 2018 that utilized a Transformer‐decoder‐only structure and continued to add modifications in later iterations.^14^, ^15^, ^16^ In just 3 years, its learning parameters exponentially grew from 110 million (GPT‐1, 2018) to 175 billion (GPT‐3, 2020). As its neural network grew increasingly intricate, the GPT model generated texts of progressively higher quality.^17^

**Fig. 2:**
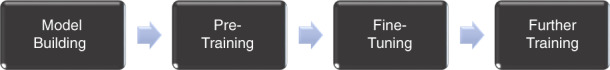
Simplified training flowchart for GPT. (a) Building the Model: Engineers in OpenAI built the basic structure of GPT called the Transformer, and set the hyperparameters (the number of layers and parameters in the Transformer), which cannot be changed by the model itself later after trained with data. (b) Pre‐Training: The model was put into the unlabeled, unsupervised pre‐training with huge amounts of data, where the model learned the patterns and presentations in languages. The learned knowledge was stored as data generated by the model, or “weights,” which could be changed after further training. (c) Fine‐Tuning: The pre‐trained model was then fine‐tuned for natural language processing tasks. Novel techniques other than fine‐tuning were also employed to acquire better performance. In the process, weights in the model were altered to better match specific tasks. The end products were the core builds of GPT called GPT‐1 to 4. Different core builds varied as their hyperparameters and the quantity of training data differed. (d) Further Training: These core builds can be further trained into even more specialized models like the chatbot ChatGPT.

The GPT model is trained in two stages. The first stage trains the model on a large corpus of unlabeled text data in a task‐agnostic, unsupervised fashion. This ‘pre‐training’ of the model consists of learning the patterns and representations in languages on its own. The second stage applies fine‐tuning and other novel training techniques such as reinforcement learning from human feedback^18^ to further train the ‘pre‐trained’ model to perform specific tasks. One of the end products was ChatGPT, a chatbot specialized in generating natural language conversations. As GPT's scale significantly increased, it also demonstrated the ability to learn new tasks well with only a few task‐specific data (‘few‐shot’ learning).^16^ Fig. 2 shows the simplified GPT training flowchart.

Despite recent improvements of the GPT model in the past few years, the GPT‐4 (the latest iteration of GPT) is reported to still have several limitations.^19^ First and foremost, GPT‐4 is not fully reliable because it sometimes “hallucinates” facts, makes reasoning errors, and even accepts obviously false statements from a user. Moreover, it can be fully confident in accepting and propagating these errors. Second, GPT‐4 suffers from a limited ability to separate facts from incorrect but statistically appealing statements. Third, GPT‐4 generally lacks knowledge of events after September 2021 because that is the end of its pre‐training data set. GPT‐4 also does not learn new knowledge from its experience. Lastly, GPT‐4 has exhibited various biases in its outputs that are well recognized. OpenAI has initiatives to minimize GPT biases in order to provide safeguards and reasonable default behaviors that reflect common community values.

## GPT and ChatGPT: Rising Stars of AIs in Psychiatry after Deep Learning

Deep learning, the principal algorithm underlying GPT, has benefited psychiatry in the recent past. Application of deep learning to classify psychiatric disorders *via* neuroimaging data is promising, especially for schizophrenia.^20^ Deep learning models based on electroencephalograms have also been created, but found to have flaws due to relatively small data sets and inexact methods.^21^ For studies using clinical data, a deep learning model using multiple patient characteristics to generate the diagnosis and prognosis of mental disorders was recently created and achieved high diagnostic accuracy.^22^

Compared to previous deep learning models, GPT has two major characteristics: (1) Natural language processing: GPT was specifically designed for NLP tasks such as text completion, generation, and classification. Its language proficiency is unique among AI models. It excels particularly in natural language generation tasks, even surpassing other pre‐trained language models such as ELMo and BERT.^23^ (2) Contextual understanding: by utilizing the self‐attention mechanism in its Transformer,^24^ GPT can process embedded information of words within sentences, and define their correlation. With these insights, GPT was trained with sentences containing masked words to predict the words based on probability (masked self‐attention).^25^ Since the training was conducted within sets of semantic contexts, it obtained a comprehensive contextual understanding and can interpret words according to context. For example, in the sentence “the cat ate a fish, and it looked happy.” GPT is capable of “understanding” the context of the latter phrase and interprets the word “it” as “the cat.” GPT also enjoyed the advantages of large‐scale training with 45 TB (GPT‐3) of data and the ability to be fine‐tuned for specific tasks, making it one of the world's most ambitious and flexible AI models. With these advantages, we see GPT's high potential to significantly expand crosstalk between psychiatry and AI. ChatGPT is the easily‐accessible chatbot version of GPT ready for immediate use in psychiatry. Here, we will illustrate some current use and limitations of ChatGPT, and present a roadmap for the future development of GPT‐based applications in psychiatry.

## Current Use and Limitations of ChatGPT for Mental Health Professionals

Due to its specialized training for chatbot language generation tasks, current ChatGPT uses in psychiatry are mainly limited to assisting psychiatrists with routine tasks. Clinical tasks of evaluation and diagnosis, assisting in psychotherapy, or patient assessments are still performed by human therapists. However, trials of ChatGPT‐assisted mental health services are being pursued. ChatBeacon, an all‐inclusive customer service platform, claimed to provide mental health assistance powered by ChatGPT. (https://www.chatbeacon.io/industry-chatgpt/mental-health-chatbot) Koko, a free therapy program, is also testing a demo of GPT‐3 mental health intervention. (https://gpt3demo.com/apps/koko-ai).

ChatGPT is ready‐made for current applications to reduce the burdens of clinical documentation, communications and research tasks.

Burnout in psychiatrists is a major issue in the profession. According to a recent meta‐analysis, the prevalence of overall burnout is as high as 50% when measured with the Copenhagen Burnout Inventory.^26^ High clinical workload and bureaucratic burdens such as arranging admissions and processing paperwork were reported as sources of burnout‐related stress in previous studies.^27^ ChatGPT may help with these burn‐out related factors. For example, ChatGPT can process transcripts of clinical dictations (automatically generated by services like Google Speech‐to‐Text) to generate summaries from medical dialogues. These can be later reviewed and revised by doctors for entry into medical records as admission notes. ChatGPT can also complete medical record documentation in standard or customized formats to reduce stressful bureaucracy and protect mental health professionals from burnout. Patel and Lam recently showed that ChatGPT can quickly and efficiently generate discharge summaries.^3^

In research, ChatGPT is renowned as an expert writing assistant. A recent experiment showed that college‐educated professionals using ChatGPT enjoyed substantially increased productivity in occupation‐specific, incentivized writing tasks.^28^ Using ChatGPT to polish later drafts of academic writing for improved readability and language is recommended. Another use of ChatGPT is to generate codes for statistical software like SAS or R. It can produce the programming backbone that is rapidly implemented after review for needed modifications. However, the current highest standards of academic ethics and rules on plagiarism should be clearly followed. Utilizing ChatGPT in research is ethically acceptable if it does not replace key researcher tasks like interpreting data and drawing scientific conclusions, as suggested by Elsevier (https://beta.elsevier.com/about/policies-and-standards/publishing-ethics?trial=true). It is important to note that according to the recommendations published by the World Association of Medical Editors (https://wame.org/page3.php?id=106), ChatGPT cannot be an author. Human authors should take full responsibility for academic work and use ChatGPT applications within current acceptable standards with transparent disclosure.

Frequently, psychiatric patients also suffer from other diseases and treating psychiatrists need to effectively and clearly communicate with other doctors and healthcare professionals. ChatGPT may facilitate this process by providing templates or polishing the contents in consultation letters and other clinical communications. In addition, ChatGPT can also improve communications between patients and psychiatrists. It was recently shown the ability of ChatGPT to rapidly and accurately generate appropriate patient clinic letters with humanity.^2^

Two significant challenges arise from the fundamental principle of ChatGPT, which is to predict words based on prompts. First, although humans can interpret the meaning of a possible complex sentence with genuine understanding and respond accordingly, ChatGPT does not. It can only generate desired results with appropriate prompts and has limited ability to improvise. It is thus important to perform potentially time‐consuming trials of using different prompts in order to learn what prompts are best suited for specific tasks. Also, since individual patients may describe their conditions in different ways during clinical encounters, the GPT structure is unlikely able to assess and respond to patients directly in clinical settings in the near future. Second, the word‐predicting nature of ChatGPT can sometimes result in generating authoritative‐sounding but incorrect output, because its training is most efficient for teaching the format rather than the content of languages. It is well documented that ChatGPT may fabricate facts and references if requested to summarize previous studies or provide an overview on an academic topic.^4^ ChatGPT's absence of clinical reasoning and accumulated experience may result in omissions of important clinical information from patient summaries and medical records. Therefore, it is highly recommended to require professionals to verify and revise ChatGPT‐generated content. However, given adequate training and further fine‐tuning, GPT has the potential to produce increasingly satisfactory and accurate results.^29^ Kahun is an evidence‐based clinical reasoning tool for physicians that recently integrated ChatGPT, and claims to have improved the practical capabilities in generating physician notes and summary letters (https://www.techtimes.com/articles/289851/20230402/pr-kahun-integrates-chatgpt-bolstering-ai-masters-fundamentals-medicine.htm). It demonstrated GPT's potential to improve *via* integration with medically trained algorithms.

In addition to the technological challenges, it will be critical to establish the professional and ethical standards of applying ChatGPT to psychiatry. With the rise of telemedicine accelerated by the COVID pandemic, it may be tempting to exploit ChatGPT for online mental health services. This would fall below professional and ethical standards due to the real possibility of patient harm due to erroneous or inappropriate ChatGPT responses. Such application should thus be closely examined further before implementation under a comprehensive, professionally accepted ethical framework.^30^ Using a simulated patient, GTP‐3 was previously tested for mental health support. In a vivid illustration of the above concerns, the GPT model unfortunately supported the patient's suggestion of suicide.^31^ Therefore, it is certainly more appropriate and ethically sound to provide telemedicine services with actual human professional participation and supervision.

## The Future: Potential Development and Challenges for GPT in Psychiatry

The capabilities of the GPT system were further enhanced with the recent release of GPT‐4.^32^ As GPT gains more power and efficiency, we foresee that there will be more momentum to further integrate and expand GPT applications in clinical psychiatry in the near future.

For complicated patient cases, GPT may provide significant assistance in generating differential diagnosis with relevant signs and symptoms and even outline an educational deductive process for training purposes at arriving at the correct diagnosis in the future. There were numerous prior reports about applying natural language processing to detect mental illness.^33^ However, these studies focused on detecting pre‐selected diseases. When training a comprehensive model to detect and classify psychiatric disorders into different diagnoses, the heterogeneous presentations of these disorders and poor reliability in psychiatric diagnosis,^34^ may bring major challenges to the training process. The current technology cannot replace the experience and judgment of expertly trained psychiatrists.

Clinical GPT usage beyond the diagnostic setting requires the development of more fundamental abilities. The clinical practice of psychiatry requires a fundamental aspect of human interaction, empathy. A therapist's empathy is an important predictor of client outcomes in psychotherapy.^35^ ‘Theory of mind’ is the origin of cognitive empathy. It was recently shown that GPT‐4 had excellent ‘theory of mind’‐like ability, as it solved 95% of false‐belief tasks.^36^ This result implied the possibility of GPT structure acquiring cognitive empathy in the future. Another fundamental skill in clinical practice is to recognize the emotion of our patients. In a recent study testing ChatGPT with affective computing tasks, ChatGPT performed well in sentiment analysis but only average on suicide assessment and poorly on personality assessment.^37^ Thus, it is plausible that ChatGPT recognizes the users' emotions with fairly good accuracy. Personality plays another important role in psychotherapy, especially for neurosis.^38^ However, a psychiatrist must undergo extensive training time and gain necessary experience to discern the full personality pattern of a patient. As shown above, ChatGPT currently lacks the ability to accurately assess personality. Nevertheless, AI experts are making technical progress to continue improving the accuracy of personality detection.^39^ Finally, the detection of mental health warning signs is an essential component of effective mental health care. Although ChatGPT's moderate performance in suicide assessment is described above, a recent study reported initial success for an AI model designed to detect cognitive distortions in text messages as accurately as clinically trained human raters.^40^ Previous research on NLP detection and prevention of suicide ideation is also yielding promise for more technological advances in AI's emotional quotient.^41^

When GPT technology is equipped with the ability to empathize, recognize emotion, assess personality, and detect mental health warning signs, we envision a future with GPT in psychiatric clinics. With full informed consent, patients can willingly provide their social media content or conversations for assessment by GPT‐based medical apps. The deduced personality profile and diagnostic workup will help psychiatrists design personalized management and treatment plans. GPT‐based medical apps may extract patients' daily conversations for evaluation of patient emotions, and provide psychiatrists with follow up data on the effectiveness of therapy plans. The ability of GPT to potentially detect mental health warning signs either in daily conversations or in text exchanges *via* telemedicine will also facilitate early and effective intervention when necessary. Empathic interactions between GPT‐based medical apps and patients may improve therapeutic adherence and efficacy. During a mental health crisis, GPT‐based medical apps may show empathic responses and reduce the risk of harm if a human mental health professional is not immediately available. Finally, with consent in user agreements or specialized app, we can potentially monitor for mental health warning signs in users' chatting with chatbots, acquaintances, and social media. These GPT‐technology‐assisted measures allow us to gather continuous health data in real time to provide patients with effective timely assistance and information, and achieve enhanced digital community mental health.

A fully‐automated psychotherapy system would be the ultimate goal for applying GPT in the field of psychiatry. Given its ability in few‐shot learning with expert communication, GPT has the potential to be utilized in training specific psychotherapy systems, such as those based on psychotherapy verbatim. However, training data must be processed to ensure the protection of privacy and to comply with all professional, ethical and legal standards. Furthermore, it is important to note that clinical reasoning cannot be trained based solely on rhetoric and requires integration with clinically‐trained algorithms. To enhance the user experience, it would be beneficial to incorporate voice receiving and generation modules, as well as friendly AI avatars as user interfaces. Furthermore, the correct evaluation of emotional states is best performed with a combination of cognitive appraisal and physiological measurements.^42^ Thus, integrating algorithms that interpret emotion‐related physiological feedback from wearable devices^43^ and facial recognition algorithms for emotion recognition may yield the most useful clinical psychiatry applications.

Despite continuous revolutionary advances in the GPT model, there are significant ethical challenges for widespread application in psychiatry and health care. One major concern derives from “Do no harm”, the principle of non‐maleficence in medical ethics. Even the advanced GPT‐4 model has potential risks of providing harmful advice.^19^ It is doubtful that we can fully eliminate these risks in the near future due to the fluid nature of language and associated training data sets. Extensive training, adjustments, and comprehensive evaluation of a fully‐automated psychotherapy system should thus be conducted before commercial release to minimize the risk of harm to patients. Supervision use of AI systems as assistants to mental health professional in providing patient care is envisioned to be the safest operational mode. If psychiatrists provide services with aid from AI systems, routine monitoring of patients and the system should be mandatory and necessary. If the AI system is noted to perform erroneously, the supervising psychiatrist must take full responsibility for any detriment done to the patient. This is the paradigm of our current training system with teams of trainees and professionals working together to deliver the best possible patient care. Like many other past technological advances introduced in health care, GPT technology is envisioned to enhance the professional team's capabilities to deliver more efficient and effective care only after much validation and real‐world testing. At the dawn of digitally‐delivered mental health care, its impact on the therapeutic alliance is still under investigation.^44^ While marching towards the revolutionary future of fully‐automated psychotherapy systems, it is essential for us to re‐examine issues such as professional and ethical standards in patient‐physician relationships, and to construct new diagnostic, therapeutic and training models that incorporate digital health care into clinical practice.

## Conclusion

Current applications of ChatGPT in mental health care are constrained by its nature as a chatbot rather than a specialized AI tool in psychiatry. Nevertheless, this advanced language‐trained model results in many useful applications for today's routine psychiatric and administrative tasks. We envision a vast potential for future GPT applications in psychiatry including GPT‐supported diagnosis, psychotherapy in clinical settings, the rapid and early identification of warning signs for suicidal tendencies, and other mental health issues in community mental health care. Most importantly, professional ethical and practice standards must be established and refined for the proper implementation of revolutionary GPT technologies in mental health care.

## Disclosure statement

The authors declare no competing interests.

## Author contributions

Conceptualization: K.‐P. S., S.‐W. C.; Project administration: K.‐P. S.; Supervision: K.‐P. S.; Writing ‐ original draft: S.‐W. C.; Writing ‐ review & editing: C.‐W. C., W.‐J. C., H.‐W. W., C.‐S. L., T. K., J. P.‐C. C., J. S. K.
